# Janus kinase inhibition prevents cancer- and myocardial infarction-mediated diaphragm muscle weakness in mice

**DOI:** 10.1152/ajpregu.00550.2015

**Published:** 2016-02-10

**Authors:** Ira J. Smith, Brandon Roberts, Adam Beharry, Guillermo L. Godinez, Donald G. Payan, Todd M. Kinsella, Andrew R. Judge, Leonardo F. Ferreira

**Affiliations:** ^1^Rigel Pharmaceuticals, South San Francisco, California;; ^2^Department of Physical Therapy, University of Florida, Gainesville, Florida; and; ^3^Department of Applied Physiology and Kinesiology, University of Florida, Gainesville, Florida

**Keywords:** critical illness, diaphragm, Janus kinase, wasting, wasting

## Abstract

Respiratory dysfunction is prevalent in critically ill patients and can lead to adverse clinical outcomes, including respiratory failure and increased mortality. Respiratory muscles, which normally sustain respiration through inspiratory muscle contractions, become weakened during critical illness, and recent studies suggest that respiratory muscle weakness is related to systemic inflammation. Here, we investigate the pathophysiological role of the inflammatory JAK1/3 signaling pathway in diaphragm weakness in two distinct experimental models of critical illness. In the first experiment, mice received subcutaneous injections of PBS or C26 cancer cells and were fed chow formulated with or without the JAK1/3 inhibitor R548 for 26 days. Diaphragm specific force was significantly reduced in tumor-bearing mice receiving standard chow; however, treatment with the JAK1/3 inhibitor completely prevented diaphragm weakness. Diaphragm cross-sectional area was diminished by ∼25% in tumor-bearing mice but was similar to healthy mice in tumor-bearing animals treated with R548. In the second study, mice received sham surgery or coronary artery ligation, leading to myocardial infarction (MI), and were treated with R548 or vehicle 1 h postsurgery, and once daily for 3 days. Diaphragm specific force was comparable between sham surgery/vehicle, sham surgery/R548 and MI/R548 groups, but significantly decreased in the MI/vehicle group. Markers of oxidative damage and activated caspase-3, mechanisms previously identified to reduce muscle contractility, were not elevated in diaphragm extracts. These experiments implicate JAK1/3 signaling in cancer- and MI-mediated diaphragm weakness in mice, and provide a compelling case for further investigation.

respiratory dysfunction is a common and serious problem in critically ill patients. Approximately one-third of critically ill patients develop respiratory failure, and the mortality rate of patients with respiratory failure is twice the rate of patients without respiratory failure (16). Inadequate ventilatory patterns and expulsive behavior limit gas exchange and airway clearance, causing pulmonary complications such as pneumonia. While the etiology is poorly understood, and likely complex and multifactorial, recent studies suggest that respiratory dysfunction in critically ill patients is due, at least in part, to diaphragm weakness. Diaphragm weakness compromises ventilatory and nonventilatory behavior (6, 7), which can contribute to increased morbidity and mortality in patients.

To begin to address this important problem, here, we investigate diaphragm muscle weakness in two distinct and highly relevant experimental models of critical illness, cancer cachexia and acute-phase myocardial infarction. Though fundamentally different in their pathogenesis, one notable commonality is elevated systemic inflammation, which is reported in critically ill human patients (2, 4), as well as preclinical models (9, 13). Experimental models characterized by systemic inflammation were selected for the current investigation because we recently found that inhibition of the inflammatory JAK 1/3 signaling pathway prevented diaphragm muscle dysfunction in a different preclinical model, mechanical ventilation (12, 14). The goal of the current investigation was to determine whether this same pathway contributes to cancer cachexia- and acute myocardial infarction (MI)-mediated diaphragm muscle weakness in mice.

## MATERIALS AND METHODS

University of Florida and Rigel Pharmaceuticals Institutional Animal Care and Use Committees approved animal experiments conducted at respective institutions. C26 cancer studies were conducted as described elsewhere (10), and MI was performed by ligation of the left anterior descending coronary artery, as previously described (1). Total and cleaved caspase-3 and actin were assessed via Western blot analysis, as explained previously (12). Statistical analysis was performed using one-way or two-way ANOVA, as appropriate, followed by Tukey's multiple comparisons test. Two-tailed *P* values of <0.05 were considered significant. Data are reported as means ± SE.

## RESULTS AND DISCUSSION

**Fig. 1.**
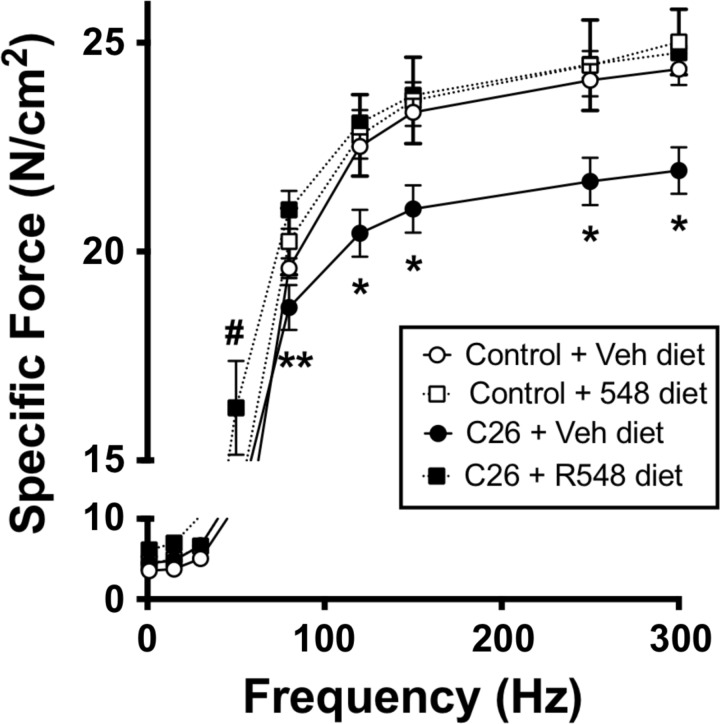
JAK 1/3 inhibition prevents cancer-mediated diaphragm weakness. Specific force-frequency relationship in diaphragm muscle strips of control mice or cachectic C26 mice fed standard chow or chow containing the JAK 1/3 inhibitor R548. Data represent means ± SE; *n* = 5 or 6 per group. *P < 0.05 C26/Veh vs. all other groups. **P < 0.05 C26/Veh vs. C26/R548. #*P* < 0.05 C26/R548 vs. all other groups.

**Fig. 2.**
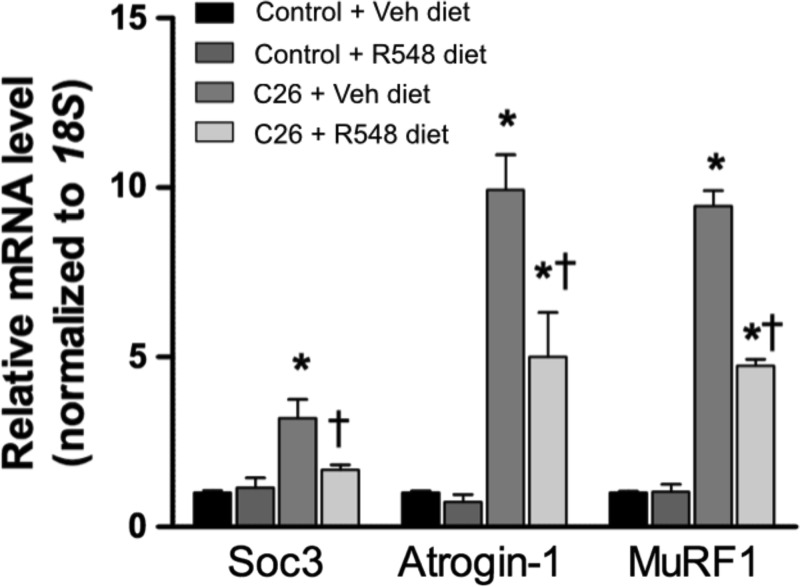
The JAK 1/3 inhibitor R548 significantly reduces messenger RNA levels of the atrogenes atrogin-1 and MuRF1 in cachectic mice. *A*: messenger RNA expression of the downstream STAT3 transcriptional target SOCS3, and the atrophy-related genes atrogin-1 and MuRF1 in TA muscle of control mice or cachectic C26 mice fed standard chow or chow containing the JAK 1/3 inhibitor R548. Data represent means ± SE; *n* = 5 or 6 per group. *P < 0.05 vs. control groups. †*P* < 0.05 vs. C26 mice fed vehicle chow.

In the first set of experiments, male CD2F1 mice received subcutaneous injections with either PBS or C26 colon carcinoma cells and were further subdivided into groups receiving either the JAK 1/3 inhibitor R548 or vehicle. R548 was formulated into chow (0.3 g R548/kg chow) and was provided to the animals upon PBS or C26 cell delivery, and throughout the study. The R548 dose was based on pharmacokinetic studies demonstrating that 0.3 g R548/kg chow maintained blood exposure levels above the levels associated with recovery of diaphragm muscle-specific force during mechanical ventilation (12, 14). Chow intake was similar between the experimental groups throughout the study, and animals were euthanized on the 26th day of the experiment. As previously reported (10), diaphragm-specific force was significantly reduced in tumor-bearing mice (*P* < 0.05; Fig. 1). Treatment with the JAK 1/3 inhibitor prevented cancer-mediated contractile dysfunction (Fig. 1), as well as diaphragm muscle wasting, which was assessed by muscle cross-sectional area (data not shown). Similarly, R548 blocked ∼80% of tibialis anterior (TA) and plantaris limb muscle atrophy in tumor-bearing mice (*P* < 0.05; data not shown) but did not prevent gastrocnemius or soleus wasting (data not shown). JAK 1/3 inhibition also significantly reduced mRNA levels of the STAT3 downstream transcriptional target SOCS3, and the atrophy-related genes atrogin-1 and MuRF1 in TA muscle of cachectic mice (Fig. 2; *P* < 0.05). Importantly, JAK 1/3 inhibition was well tolerated in tumor-bearing mice and did not exacerbate tumor growth or whole body cachexia. These data identify JAK 1/3 signaling as a key mediator of diaphragm muscle weakness during cancer cachexia in mice and establish inhibition of JAK 1/3 signaling as a promising therapeutic strategy worthy of additional investigation.

**Fig. 3.**
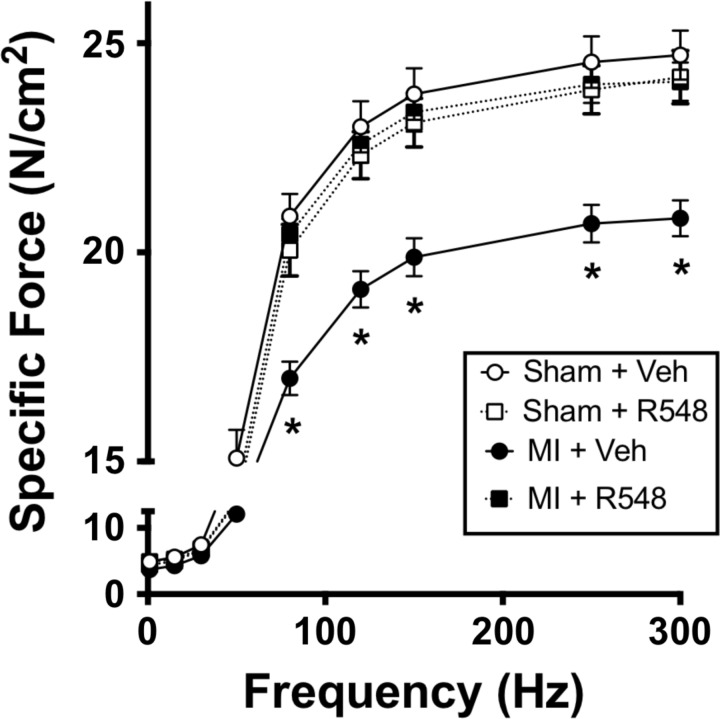
Myocardial infarction-induced diaphragm weakness is prevented by JAK 1/3 inhibition. Specific force-frequency relationship in diaphragm muscle strips of sham-operated or mice subjected to myocardial infarction and treated with vehicle or R548 1 h postsurgery, and once daily for 3 days. Data represent means ± SE; *n* = 6–8 per group. *P < 0.05 MI/Veh vs. all other groups.

**Fig. 4.**
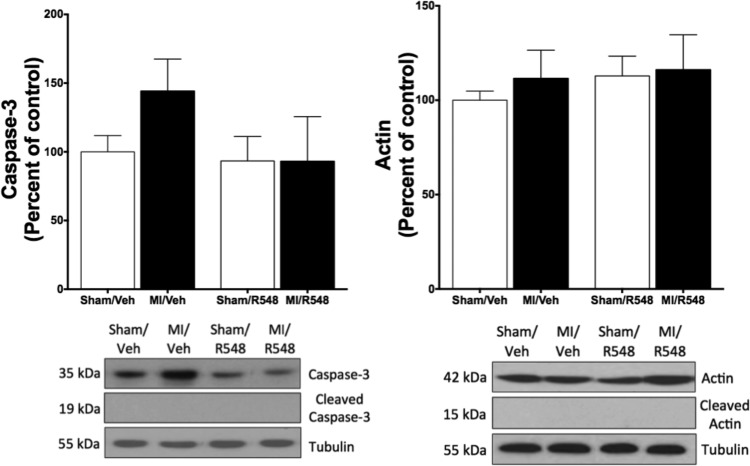
Assessment of caspase-3 activation in diaphragm muscle 72 h after myocardial infarction. Total and cleaved caspase-3 (*left*) and actin (*right*) protein levels assessed via Western blot analysis. Data represent means ± SE; *n* = 6–8 per group.

In the second series of experiments, male C57BL/6 mice were randomly assigned to one of four groups: *1*) sham surgery/vehicle treatment, *2*) sham surgery/R548 treatment, *3*) myocardial infarction/vehicle (MI/vehicle), and *4*) MI/R548. MI was initiated by ligation of the left anterior descending coronary artery, and the JAK 1/3 inhibitor was delivered 1 h post-MI (75 mg/kg body weight sc) and continued once daily for 3 days. Pharmacokinetic studies were conducted to determine the subcutaneous dose required to maintain plasma levels of the active drug above the efficacious level identified during mechanical ventilation (12, 14). Seventy-two hours after MI, diaphragm muscle-specific force was diminished by nearly 20% (*P* < 0.05), and therapeutic administration of R548 completely prevented this loss (Fig. 3). Measurements of cardiac pathology, such as mean left ventricular weight and epicardium infarct area, were unchanged with JAK 1/3 inhibition (data not shown), arguing that improved diaphragm function was likely related to intrinsic factors within diaphragm muscle. Inflammation-mediated reactive oxygen species and oxidative stress may reduce muscle-specific force after acute MI (3); therefore, we next determined whether MI increased cellular markers of oxidative damage. However, protein carbonyl and 4-HNE levels, markers of oxidative stress assessed in whole muscle lysates, were not significantly elevated in diaphragm muscle from MI mice (data not shown). We also evaluated caspase-3 activation, since previous studies suggest that this apoptotic protease contributes to muscle wasting and weakness (5, 11) and because we previously showed that JAK 1/3 inhibition during mechanical ventilation prevented activation of caspase-3 (12). Western blot analysis revealed no significant differences in the protein levels of total or cleaved (activated) caspase-3 (Fig. 4). Additionally, there were no significant differences in the amount of partially degraded (cleaved) actin, an established caspase-3 proteolytic substrate (Fig. 4). Together, these data suggest that caspase-3 was unlikely to have contributed to diaphragm weakness at the time point evaluated here. While markers of oxidative damage in whole cell lysates were unchanged in the current study, oxidative modifications to specific contractile proteins following MI, as was recently reported (3), may have contributed to diminished diaphragm force. Indeed, we previously reported that inhibition of JAK/STAT3 signaling significantly reduced mitochondrial dysfunction and oxidative damage in diaphragm muscle during mechanical ventilation (12). This study demonstrates for the first time prevention of diaphragm weakness following acute myocardial infarction using a clinically relevant drug treatment regimen. Additionally, our findings identify the important role of JAK 1/3 signaling in MI-induced diaphragm weakness in mice.

Systemic inflammation is common among patients with cancer and acute MI and is also reported in experimental models of these conditions. Of the proinflammatory cytokines typically upregulated in these critical illnesses, preclinical studies link the IL-6/JAK/STAT3 signaling pathway to muscle wasting and dysfunction. In this pathway, binding of the IL-6/IL-6 receptor complex to the transmembrane glycoprotein 130 (gp130) leads to phosphorylation and activation of the intercellular JAKs and their downstream target STAT3 (8). Activated STAT3 translocates into the nucleus and initiates target gene transcription. Recent preclinical studies demonstrate the important role of the IL-6/JAK/STAT3 pathway in muscle wasting and weakness in a variety of conditions, and further show diverse mechanisms connecting the pathway to muscle atrophy and dysfunction. For example, as mentioned above, mechanical ventilation in rats resulted in STAT3 accumulation within mitochondria, mitochondrial dysfunction, elevated oxidative damage, and diaphragm weakness, which were prevented by JAK 1/3 inhibition (12). In aged mice and mice with muscular dystrophy, STAT3 activation blunted satellite cell expansion and inhibited muscle repair following injury (15). In an experimental model of chronic kidney disease, STAT3 initiated muscle wasting through activation of the transcription factor C/EBPδ, and a potent negative regulator of muscle mass, myostatin (17). Similar findings were reported in preclinical models of cancer cachexia (11). Here, we expand on previous studies and report for the first time the critical role of JAK 1/3 signaling in diaphragm muscle weakness in animal models of cancer cachexia and acute MI. Improved diaphragm contractility throughout the force frequency curve suggests that JAK 1/3 inhibition in these preclinical models may improve low- to moderate-intensity diaphragm function typically utilized at rest and during normal activity, as well as maximal force required during high respiratory demand. Further studies are necessary to better understand the precise mechanisms of JAK 1/3-mediated improvements in diaphragm-specific force in these experimental models, as well as the potential impact on respiratory function.

In conclusion, our findings highlight the important role of JAK 1/3 signaling in diaphragm muscle dysfunction in both cancer cachexia and acute MI in mice and provide a promising therapeutic target to preserve diaphragm function in these circumstances. These findings may have relevance to respiratory muscle dysfunction in critical illness in humans and warrant further study.

## DISCLOSURES

I. J. Smith, G. L. Godinez, D. G. Payan, and T. M. Kinsella are employees and/or stockholders of Rigel Pharmaceuticals.

## AUTHOR CONTRIBUTIONS

Author contributions: I.J.S., D.G.P., T.M.K., A.R.J., and L.F.F. conception and design of research; I.J.S., B.R., A.B., G.L.G., A.R.J., and L.F.F. performed experiments; I.J.S., G.L.G., A.R.J., and L.F.F. analyzed data; I.J.S., T.M.K., A.R.J., and L.F.F. interpreted results of experiments; I.J.S., B.R., A.B., A.R.J., and L.F.F. prepared figures; I.J.S. drafted manuscript; I.J.S., D.G.P., T.M.K., A.R.J., and L.F.F. edited and revised manuscript; I.J.S., T.M.K., A.R.J., and L.F.F. approved final version of manuscript.
